# Ionocyte Immunolocalization and the Effects of Ultraviolet Radiation on Their Abundance and Distribution in the Alenins of Caspian Sea Salmon, *Salmo trutta caspius*

**Published:** 2011-04-21

**Authors:** Ensiyeh Ghanizadeh Kazerouni, Saber Khodabandeh

**Affiliations:** Marine Biology Department, Tarbiat Modares University (International Campus), Noor, Iran

**Keywords:** UV Radiation, *Salmo trutta caspius*, Skin, Gills, Immunolocalization

## Abstract

**Objective:**

On a global scale, stratospheric ozone depletion has caused an increase in UV-B radiation reaching the earth's surface. Ultraviolet radiation has long been suspected to be harmful to aquatic organisms.

**Materials and Methods:**

In order to study ionocyte localization (by Na^+^/K^+^-ATPase immunolocalization) and the effects of UV radiation on the ionocytes of skin and gills, the alevins of *Salmo trutta caspius* were exposed to different doses of UV radiation [unit low doses (ULD) of: 60 µw/cm^2^ UVC; 100 µw/cm^2^ UVB and 40 µw/cm^2^ UVA and unit high doses (UHD) of: 90 µw/cm^2^ UVC; 130 µw/cm^2^ UVB and 50 µw/cm^2^ UVA] using two adjustable F8T5 UV-B, 302 nm lamps (Japan) for 15 minutes once a day in laboratory conditions. Alevins not subjected to UV exposure served as a control group.

**Results:**

In both UV exposure groups, all the alevins died on the ninth day. No mortality was observed in the control group. The Na^+^/K^+^-ATPase immunolocalization study indicated that ionocytes were located, in lessening order, on the yolk sac, trunk, gills, opercula and rarely on the head skin. Immunohistochemical results showed significant reduction in the number of ionocytes on the yolk sac, with lesser reduction on the trunk in both UV exposure groups. In contrast, the number of immunofluorescence cells on the gill was significantly elevated. Our results also showed that the size of ionocytes was reduced on the trunk and yolk sac in the UV exposure groups, but not significantly. Deformation and destruction of ionocytes on the yolk sac and trunk were observed with scanning electron microscope (SEM) in the UV exposure groups.

**Conclusion:**

Our results showed that ionocytes were located mainly on the yolk sac, in lesser amounts on the trunk, gills and opercula, and rarely also on the head skin of alevins. UV radiation caused deformation and reduction in the number and size of ionocytes on the trunk and yolk sac. As the skin cells of trout alevins possess essential functions for respiration, osmoregulation, excretion and defense during this stage of life, the observed damage may have contributed to their suddenly mortality in the UV exposure condition.

## Introduction

Ion exchanges occur in specialized cells, namely chloride cells or mitochondria-rich cells (MRCs), or more generally ion-transporting cells or ionocytes (1, 2). Ionocytes, possessing a high content of Na^+^/K^+^-ATPase, regulate the body fluid content in fish (osmoregulation). Ultrastructurally, ionocytes are large in size, with apical microvilli or an apical pit and numerous mitochondria associated to basolateral membrane infoldings (2). Many studies have shown the sites of osmoregulation and ionocytes in adult fish to include gills, intestines, kidneys and rarely, skin (2-7). However few studies exist on ionocyte distribution and osmoregulation in the early stages of fish development. Although fish larvae also need to osmoregulate, adult osmoregulatory organs are underdeveloped or absent in these early developmental stages. Thus the skin is the main site of osmoregulation, and ionocytes are located on the trunk, yolk sac, head, and fins (2, 8-10). In a review publication it is demonstrated that ionocyte abundance and distribution can differ between different species of fish (2).

The skin of newly hatched teleost larvae has several important physiological functions. It is involved in osmoregulation, respiration and excretion. In order for efficient respiratory and osmoregulatory exchanges to be possible, it is essential that the epidermis be thin enough to allow gas and ion exchanges. The importance of the skin in early developmental stages is also owing to the fact that the surface to volume ratio is high in the early stages and decreases during the course of development (2). However, these characteristics of skin in larvae make them vulnerable to the harmful effects of UV radiation.

The level of UV-B radiation on the earth’s surface has increased because of the progressive depletion of the ozone layer in recent years (11). This depletion is expected to continue for several decades due to the synergistic effects of ozone depletion and global climate changes (12). Although UV-B radiation represents a small part of the solar spectrum, it gives rise to many biological and photochemical processes that are quite harmful to organisms (13, 14). It has been reported that long-term exposure of humans to UV-B radiation may have very serious consequences inducing skin cancer, cataracts and a dangerous weakening of immune functions (15). Therefore scientists have become interested in the biological consequences of long-term exposure to UV-B radiation in aquatic organisms. Today, the detrimental effects of UV-B radiation on aquatic ecosystems are well documented (16-18). It has been shown that present and predicted UV-B levels for the next decade can increase the mortality of phytoplankton, zooplankton, large invertebrates such as corals and anemones, amphibians and fishes, especially those with planktonic larvae (19-21). However, although a substantial amount of researches has been done on fish, the effects of UV-B radiation on the development of freshwater species are largely unexamined (22).

The effects of UV-B radiation on the skin of freshwater fish are particularly interesting for the following reasons. Fish epidermis is a naked, non keratinized epithelium with no external protection against irradiation. Fish skin has photo protective products, but those are located immediately below the epidermis, so the epidermis is more sensitive in fish than in mammals, in which it contains epidermal melanosomes (23, 24). Furthermore, the eggs and larvae of fish are most susceptible to UV-B damage (16, 25). This is partly because, as mentioned above, larvae have a high surface area to volume ratio which increases the proportion of cells at or near the external surface. In addition, eggs and larvae tend to have low concentrations of photo-protective compounds because the synthesis of pigments such as melanin is induced by exposure to light, and mycosporine-like amino acids can only be acquired through consumption. Eggs and larvae also have limited behavioral capabilities to avoid UV-B exposure due to their reduced mobility, and some species cannot detect UV-B radiation in the early developmental stages (12).

In this study we investigated the distribution of ionocytes by immunolocalization of Na^+^/K^+^-ATPase and the effects of artificial UV radiation on ionocytes in the alevins of Caspian trout Salmo trutta caspius. Salmonids are fish species with a commercial interest, predominantly inhabiting clear waters which allow greater penetration of UV-B radiation than turbid or colored waters. Several studies report the histological and histopathological alterations in the skin of salmonid fish following exposure to solar or artificial UV-B radiation, including the changes in the quantity and location of epidermal mucous cells, advanced spongiosis and edema, extensive hyperplasia as well as open ulcers and eroded fins (23, 24, 26). UV-B exposure affects the mechanism of wound repair in salmonids skin (27) and increased susceptibility to fungal diseases and higher mortality rates have also been reported (11, 26). The loss of epidermal integrity due to solar radiation facilitates the entry of pathogens and leads to osmotic disturbance (28). It has also been hypothesized that solar radiation may be partly responsible for the lower survival rates and recruitment of anadromous salmonids (29, 30). *Salmo trutta caspius* is an anadromous subspecies of brown trout restricted to the Caspian Sea, particularly the southern margin along the Iranian coast (31). Stock of this highly precious species has so drastically decreased that it has been enlisted as an endangered species (32). There are several other reasons for increased UV penetration, such as the decrease in river flow rate and depth due to the construction of dams. The natural solar UV intensity in their spawning area was 40 µw/cm^2^ of UVC; 90 µw/ cm^2^ of UVB and 190 µw/cm^2^ of UVA in the 40 cm depth of water near the naturally spawned eggs at 12 o’clock (26). The present investigation aims to evaluate the UV sensitivity of the Caspian trout alevins in laboratory conditions, through the study of ionocytes abundance and distribution changes on the skin and in the gills, by immunolocalization and ultrastructural observation.

## Materials and Methods

### Fish

Fertilized eggs (two days to hatch, 5 mm diameter) of *S. trutta caspius* were collected from the aquaculture center of Shirudi at Kelardasht (Iran) in the winter of 2007 and kept in a 40L tank at 9℃ in the aquaculture laboratory of Tarbiat Modares University. Following a 12 hour adaptation period to laboratory conditions, they were randomly divided into three different groups (350 eggs in each group), and transferred to the experiment tank. One hatchery tank (60 cm × 300 cm) was divided into three units and each unit (60 cm × 100 cm) was divided into three subunits. 350 eggs were transferred to each unit and each unit was covered with a black plastic sheet, which prevented reception of any solar light by eggs. Culture conditions were kept similar in all units (pH= 8.4, conductivity = 51.1 µs/cm, temperature = 10 ± 2, dissolved oxygen = 7.7 mg/L)

### Ultraviolet radiation

In natural condition, the solar UV intensity was 40 µw/cm^2^ of UVC; 90 µw/cm^2^ of UVB and 190 µw/cm^2^ of UVA in the 40 cm depth of water near the naturally spawned eggs at 12 o’clock. To reach a dose of UV that would not be lethal in the early developmental stages and also similar to the dose of natural solar radiation, various irradiation intensities and periods were preliminarily tested. Finally, a unit not exposed either to solar light or UV radiation served as a control group, and two UV exposure units received two different doses [unit low doses (ULD) of: 60 µw/cm^2^ UVC; 100 µw/cm^2^ UVB and 40 µw/cm^2^ UVA and unit high doses (UHD) of: 90 µw/cm^2^ UVC; 130 µw/ cm^2^ UVB and 50 µw/cm^2^ UVA], using two adjustable F8T5 UV-B, 302 nm lamps (Japan). Eggs (5 mm diameter) and alevins (19-20 mm) were exposed to UV radiation everyday for 15 minutes (from 13:00 to 13:15) for 9 days. The depth of water was 8 cm. Radiation values in µw/cm^2^ were obtained by the use of a UVX radiometer (UVP, USA) using three UV sensors (UVX-25, UVX-31 and UVX-36) (26).

### Immunolocalization study

Immunolocalization of Na^+^/K^+^-ATPase was performed through immunofluorescence light microscopy using a mouse monoclonal antibody, IgGα5, raised against the α-subunit of chicken Na^+^/K^+^-ATPase obtained from the Developmental Studies Hybridoma Bank, developed under the auspices of the National Institute of Child Health and Human Development (NICHD) and maintained by the University of Iowa (USA) (1, 3, 33). The α5 monoclonal antibody recognizes all 3 isoforms of the α-subunit of Na^+^/K^+^-ATPase in invertebrates, where they are present. This antibody is able to cross-react with the α-subunit of invertebrate Na^+^/K^+^-ATPase. Following 24 hours in Bouin’s fixative and embedment in paraffin, 4 µm sections (whole body) were cut on MICRODS 4055 microtome and collected on poly-L-lysine coated slides. Sections were first hydrated and so washed for 10 minutes in phosphate buffered saline (PBS) (15 PBS pill at 1500 cc water), 10 minutes in PBS2 (200 cc PBS1 + 1.78 mg NaCl + 40 µl Tween 20) and 20 minutes PBS3 (200 cc PBS1 + 10 mg Regiler). The primary antibody diluted in PBS4 (20 cc PBS3 + 180 cc water),(50% antibody + 50% PBS4) was placed on the sections and incubated for 2 hours at room temperature in a moist chamber. The sections were then incubated for one hour in the secondary antibody fluorescein isothiocyanate conjugate (FITC) in dark condition. The slides were rinsed in PBS and mounted in a medium for fluorescent microscopy to retard photo-bleaching. Negative control sections were incubated in a PBS series without the primary antibody (3, 33). A Nikon digital camera, adapted to the Nikon fluorescent microscope, was used to obtain images from tissues.

### Scanning Electron Microscopic study

For Scanning Electron Microscopy (SEM), 6 samples from each group were placed in cold 4% glutaraldehyde in 0.1 M phosphate buffer, pH=7.4, containing 5% sucrose, every 48 hours for the duration of the experiment. After an initial 1 hour fixation, followed by rinses in 0.1 M phosphate buffer containing 5% sucrose, the samples were post-fixed for one hour in 2% osmium tetra oxide in 0.1 M phosphate buffer, pH=7.4, containing 5% sucrose. Samples were rinsed in buffer and then several times in distilled water and were dehydrated in a graded series of ethanol. They were dried and coated with gold-palladium in a coater sputter SCDOOS (Bal-TEC Swiss). Samples were examined and photographed using a XL30 Scanning Electron Microscope at 15 kV (26, 34).

### Statistical analysis

The effects of UV radiation on the number of ionocytes per 1000 µm^2^ of skin and gills were tested by image tools; EXCEL, one-way ANOVA and Tukey’s test.

## Results

**Fig 1:**
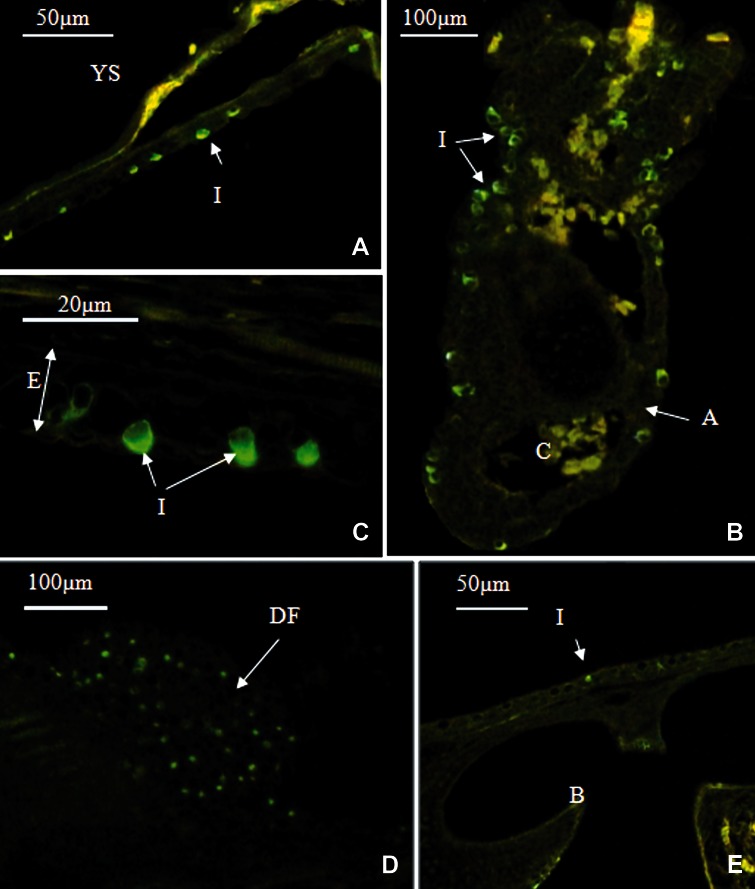
Ionocyte localization (by immunolocalization of Na^+^/K^+^-ATPase) in the skin and gill of Salmo trutta caspius in the control group on the first day. (1A): The majority of ionocytes were observed on the yolk sac. (1B): ionocytes were observed on gill arc and the lamellae had not developed yet. (1C): Some ionocytes located on dorsal part. (1D): On the trunk, ionocytes mostly located on fins. (1E): Almost no ionocytes on the head skin. A, Gill Arc; F, Gill Filament; E, Epidermis; IC, Ionocyte; YS, yolk sac; DF, dorsal fin; B, brain.

No significant mortality was observed in the control group during the course of the experiment. For both UV radiation groups, mortality level was low during the first days, but suddenly increased on the sixth day until all of the alevins had died on the ninth day. The immunolocalization study showed that ionocytes were exhibited on the yolk sac (Fig 1A), gill (Fig 1B), trunk (Fig 1C), fin bud (Fig 1D) and opercular membrane (not shown). No specific immunofluorescence staining was observed on the head skin (Fig 1E).

In the control group, on the first day, the majority of ionocytes were located on the yolk sac, followed by the trunk and gill (Table 1).

**Fig 2:**
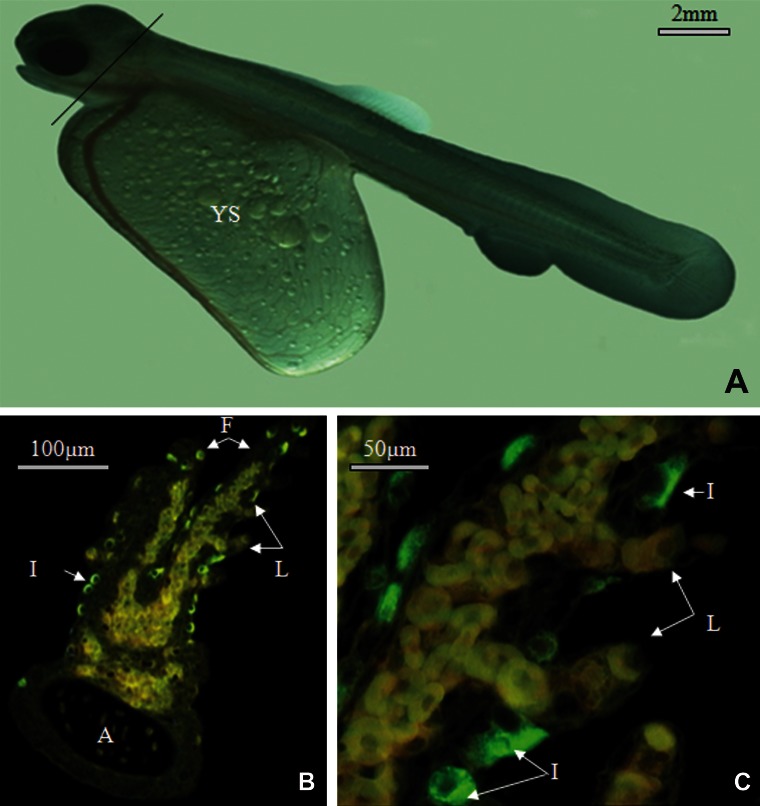
Ionocyte localization (by immunolocalization of Na^+^/K^+^-ATPase) in the gill of Salmo trutta caspius in the control group on the seventh day. (2A): 7 day old Salmon trutta caspius alevins. (2B) and (2C): ionocytes were observed on gill filaments at the base of lamellae. A, Gill Arc; F, Gill Filament; IC, Ionocyte; L, gill lamellae.

High densities of these cells were found at anterior and posterior ends of the yolk sac, where the integument covers the pericardial membrane and a vessel network near the anal opening, respectively. On the trunk, the ionocytes were mainly present on the fins (Fig 1D), but were distributed near-randomly in other parts. In the gill, the ionocytes were mostly seen on the arcs and partly on filaments (Fig 1B). On the third day, the number of ionocytes on the trunk and yolk sac had decreased but the majority of ionocytes were observed on the yolk sac, followed by gill and trunk. The number of ionocytes on the yolk sac was significantly higher than on the gill and trunk (Table 1). On the fifth, seventh and ninth days, the trend of reduction of ionocytes on the yolk sac and trunk continued and strong immunoreactivity was observed in the large spherical cells (ionocytes) located on the gill, and to a lesser extent on the yolk sac and trunk. During these days the number of ionocytes on the gill was significantly higher than on the yolk sac and trunk (Table 1). On the gill, at this time, lamellae appeared, so immunofluorescence cells were shown on filament at the base of lamellae (Fig 2A, B, C).

**Table 1:** The ionocyte numbers in gill, trunk and yolk sac of Salmo trutta caspius alevins, in the control group during the experimental period


Days	1	3	5	7	9

Gill	1.4 ± 0.23a	2 ± 0.3^a^	2.4 ± 0.23^b^	2.9 ± 0.25^b^	3.24 ± .55^b^
Trunk	1.65 ± 0.16^a^	1.4 ± 0.16^a^	1.4 ± 0.1^a^	1 ± 0.13^a^	0.8 ± 0.16^c^
Yolk sac	2.75 ± 0.3^b^	2.4 ± 0.35^b^	1.9 ± 0.3^a^	1.75 ± 0.18^a^	1.45 ± 0.13^a^


Values are given as the Mean ± SD of the number of ionocytes in 1000 µm^2^. Different letters and numbers indicate the level of significance (p<0.01).

**Table 2:** Relative changes of ionocyte numbers in the gill of Salmo trutta caspius alevins, in the control group and two UV exposure groups, during the experimental period. (UV-ULD): unit low doses of UV, (UV-UHD): unit high doses of UV


Days	1	3	5	7	9

Control	1.4 ± 0.23^a^	2 ± 0.3^a^	2.4 ± 0.23^b^	2.9 ± 0.25^b^	3.24 ± .55^c^
UV-ULD	1.46 ± 0.28^a^	2.3 ± 0.4^b^	3 ± 0.26^b^	3.17 ± 0.2^b^	3.7 ± 0.26^c^
UV-UHD	1.3 ± 0.23^a^	1.8 ± 0.3^a^	2.3 ± 0.4^b^	2.9 ± 0.33^b^	3.6 ± 0.18^c^


Values are given as the Mean ± SD of the number of ionocytes in 1000 µm^2^. Different letters and numbers indicate the level of significance (p<0.01).

**Table 3:** Relative changes of ionocyte numbers in the trunk of Salmo trutta caspius alevins, in the control group and two UV exposure groups, during the experimental period. (UV-ULD): unit low doses of UV, (UV-UHD): unit high doses of UV


Days	1	3	5	7	9

Control3	1.65 ± 0.16^a^	1.4 ± 0.16^a^	1.4 ± 0.1^a^	1 ± 0.13^a^	0.8 ± .16^b^
UV-ULD	1.5 ± 0.22^a^	1.2 ± 0.12^a^	0.85 ± 0.18^b^	0.6 ± 0.06^b^	0.4 ± 0.04^b^
UV-UHD	1.7 ± 0.5^a^	1.3 ± 0.13^a^	0.7 ± 0.4^b^	0.45 ± 0.05^b^	0.3 ± 0.18^b^


Values are given as the Mean ± SD of the number of ionocytes in 1000 µm^2^. Different letters and numbers indicate the level of significance (p<0.01).

**Table 4:** Relative changes of ionocyte numbers in the yolk sac of Salmo trutta caspius alevins, in the control group and two UV exposure groups, during the experimental period. (UV-ULD): unit low doses of UV, (UV-UHD): unit high doses of UV


Days	1	3	5	7	9

Control	2.75 ± 0.3^a^	2.4 ± 0.36^a^	1.9 ± 0.3^b^	1.75 ± 0.18^b^	1.45 ± .13^b^
UV-ULD	3 ± 0.25^a^	2.3 ± 0.5^a^	1.4 ± 0.45^b^	0.9 ± 0.2^c^	0.7 ± 0.23^c^
UV-UHD	2.9 ± 0.19^a^	2.3 ± 0.14^a^	1.1 ± 0.21^b^	0.7 ± 0.18^c^	0.45 ± 0.19^c^


Values are given as the Mean ± SD of the number of ionocytes in 1000 µm^2^. Different letters and numbers indicate the level of significance (p<0.01).

**Fig 3:**
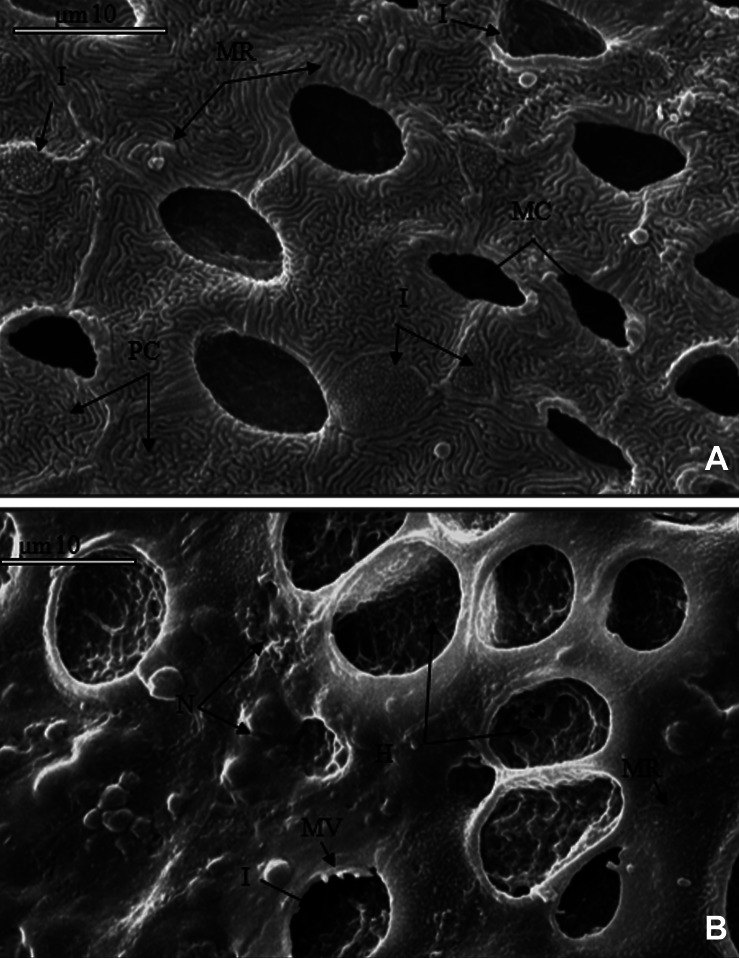
SEM micrograph of the dorsal part of skin in 9 day alevins from the control group (3A) and high dose (UHD) exposure groups (3B). (3A): apical pit of ionocytes (microvili without pit (I) and pit (II)) and mucous cells (deep pit without any ridge) located among pavement cells. Mucus secretions were also seen. (3B): skin severely destroyed and apical pit of ionocytes and mucous cells are deformed; pavement cell microridges were hardly observable. Many holes induced by mucus secretion are present. MC, Mucous cells; IC, Ionocyte; PC, Pavement cells; MR, Micro-ridge; H, Hole induced by mucous secretion; B, Boundary; MV, Microvili; N, Necrosis.

SEM micrographs of the epidermis in the control group showed the ionocytes (IC), mucous cells (MC) and mucus secretions placed between pavement cells (PC). The boundary of pavement cells which contain well-developed micro-ridges was clearly demarcated (Fig 3A).

In both UV exposure groups, immunolocalization study revealed that the first day after being exposed to UV radiation, no significant change in the number of ionocytes in different parts of the body was observed. At this time the number of ionocytes on the gill, yolk sac and trunk were almost the same as in the control group (Tables 2, 3, 4).

Following UV exposure on the fifth, seventh and ninth days, the number of immunofluorescence cells increased on the gill and decreased on the yolk sac and trunk (Tables 2, 3, 4). The difference in the reduction of ionocytes on the trunk was significant between UV exposure groups and the control group on the fifth and seventh days, but insignificant on the ninth day. On the yolk sac, reduction was insignificant on the fifth but significant on the seventh and ninth days. There was no significant difference between the two UV exposure groups. The increase in the number of ionocytes on the gill was not significant when comparing the control and UV exposure groups. Results also showed that on the ninth day, the size of ionocytes was reduced in the gill, trunk and yolk sac in UV exposure groups but not significantly (Table 5).

**Table 5:** Relative changes of ionocyte size in the branchia, trunk and yolk sac of Salmo trutta caspius alevins, in the control group and two UV exposure groups, on the 9th day. (UV-ULD): unit low doses of UV, (UV-UHD): unit high doses of UV


	Control	UV-ULD	UV-UHD

Gill	54.7 ± 2.3^a^	53.25 ± 4.1^a^	52.6 ± 4.3^a^
Yolk sac	52.4 ± 3.1^a^	52.34 ± 3.6^a^	47.82 ± 2.3^a^
Trunk	53.37 ± 2.4^a^	52.14 ± 2.8^a^	50.36 ± 4.2^a^


SEM micrographs revealed no damage to the ionocytes on the yolk sac and trunk by the first and third days (not shown). After more days of UV exposure, the deformation and destruction of skin cells consisting of ionocytes had begun, and was at its peak on the ninth day (Fig 3B).

## Discussion

We observed that, in *Salmo trutta caspius* alevins, ionocytes are distributed on the yolk sac, gill, trunk, fin bud and opercular membrane. In the control group, on the first and third days, there was a higher density of ionocytes on the yolk sac than on the gill and trunk. On the yolk sac, the density of ionocytes was higher in the anterior and posterior parts where the yolk sac’s skin touches the trunk skin. Gill ionocytes were mostly located on arcs because lamellae had not yet developed. On the trunk, ionocytes were chiefly located on the fins because the epidermis is at its thinnest on these parts. The distribution of ionocytes on other parts of the trunk was random. With the growth of the fish, on the fifth, seventh and ninth days ionocytes were mostly apparent on the gill, followed by the yolk sac and trunk. At this time lamellae had grown and ionocytes were observed on the filament at the base of lamellae.

Previous studies have also shown that in the first hatched fish, ionocytes were distributed on different parts of the skin (2, 35). In tilapia *O. mossambicus* larvae (9) skin ionocytes were found on the head, yolk sac, opercular membrane and in the buccal cavity. As in our results on *Salmo trutta caspius* in tilapia, local high densities of ionocytes were found at the anterior and posterior ends of the yolk sac (9). Wales and Tytler reported high concentrations of ionocytes on the head, yolk sac and trunk of larval herring *C. harengus* 24 hours after hatch (10). In sea bass *D. labrax* (35), large ionocytes are randomly distributed all over a wide circulatory space covering the ventral and lateral sides and particularly the yolk sac. However, a study on newly hatched larvae of *Cyprinus carpio* revealed only a few ionocytes on the surface of the yolk sac and none were found anywhere else (28). Also in the stenohaline seawater flounder *Kareius bicoloratus* (36), numerous ionocytes were found on the inner surface of the gill chambers and skin near the openings of the bronchial chambers. Only a few ionocytes were observed in other regions of the skin. As reported in many other teleosts, following yolk sac absorption and gill development in Caspian salmon alevins, the osmoregulatory function shifts from the skin to the gills, which then become the main osmoregulatory site.

We have not found investigation on the effects of UV radiation on fish ionocytes but some studies have examined the UV effects on fish skin cell structure and ultrastructure (10, 18, 26). Ionocytes are one type of skin cell that can also be affected by UV radiation. Using immunolocalization of Na^+^,K^+^-ATPase α-subunit and vital staining, in the present study, we considered changes in the trend of ionocytes on the skin and gills in control group and UV exposure alevins.

Our immunolocalization results revealed that UV radiation significantly reduced the number of ionocytes on the yolk sac. This reduction was significant on the seventh and ninth days of the experiment. However, the decline of ionocytes on the trunk was less remarkable. Seeing as the yolk sac is large at this stage and can be exposed to UV radiation more than other parts of the skin, this may explain the greater reduction of ionocytes on the yolk sac. Our experiment did not show considerable changes in the number and size of ionocytes on the gill. The operculum covers the gill cavity, arcs, filaments and lamellae, which may be one reason for the lesser effect of UV radiation on the number and size of gill ionocytes.

Studies of other pollutant effects on fish ionocytes have shown that an increase in both the number and size of chloride cells was the most obvious and constant characteristic in gill sampled at pH=6.2 to 6.5. This phenomenon is well documented and has been described in various fish species under different conditions (37). According to Mallat the increase of chloride cells has to be considered largely non-specific in nature and may primarily represent a stereotyped physiological reaction (38). In contrast, Parashar and Banerjee, with an experiment on the gills of air-breathing catfish *Heteropneustes fossilis*, exhibited that lethal concentrations of lead nitrate induced periodical fluctuations in their density at different stages of exposure. Generally, the density of these ionocytes decreases, invariably in the thickened epithelial (39).

Our SEM micrograph observation in the UV exposure groups showed that exposure to UV for three days did not have remarkable effects on skin structure. Further exposure to UV radiation was associated with deformation of ionocytes and necrosis, which were at their peak on the ninth day. Sharma et al. (10) found that the exposure of ayu, Plecoglossus altivelis, larvae to UV-B radiation (1/4 w/m^2^ for 30 days) destroyed the microridges in the epidermis and subsequently exposed neuromast cells of skin. Study on the gills of Catla catla following UV-B exposure showed the same pavement cell damage (18). There is no SEM study specifically on the UV effects on ionocytes but since the pavement cells which enclose ionocytes have been considered an aid to osmoregulation, damage to them can be lethal for Salmo trutta caspius alevins.

## Conclusion

We concluded that ionocytes were located mainly on the yolk sac and in lessening order the trunk, gills and opercula, and also rarely on the head skin of alevins. As it is directly exposed to UV radiation, skin can be the primary endangered organ in the aqueous exposure of larval fish, which involve their skin in respiration and osmoregulation during the early developmental stages. The study conducted here showed that although species that live in shallow transparent water protect their skin by synthesizing UV-B absorbing substances, even UV-B resistant fish suffer serious damage to their skin. This includes severe damage in terms of necrosis and reduction in the number and size of ionocytes on the yolk sac, which is the main site of osmoregulation for newly hatched larval, leading to deformation of the apical pit and failure of the osmoregulation function in larval. These damages may be involved in the sudden mortality in larvae of *Salmo trutta caspius* after exposure to UV radiation.

## References

[1] Khodabandeh S, Taghizadeh Z (2006). Immunolocalization
of Na^+^/K^+^-ATPase and ionocytes in gills of catfish,
Silurus glanis. Yakhteh.

[2] Varsamos S, Nebel C, Charmantier G (2005). Ontogeny
of osmoregulation in postembryonic fish: a review. Comp Biochem Physiol A Mol Integr Physiol.

[3] Khodabandeh S, Shahriari Moghaddam M, Abtahi B (2009). Changes in chloride cell abundance, Na^+^,K^+^-ATPase
immunolocalization and activity in the gills of golden
grey mullet, Liza aurata, fry during adaptation to different salinities. Yakhteh.

[4] Witters H, Berckmans P, Vangenechten C (1996). Immunolocalization
of Na^+^, K^+^-ATPase in the gill epithelium of
rainbow trout, Oncorhynchus mykiss. Cell Tissue Res.

[5] Khodabandeh S, Khoshnood Z, Mosafer S (2009). Immunolocalization
of Na^+^, K^+^-ATPase-rich cells in the gill
and urinary system of Persian sturgeon, Acipenser
persicus, fry. Aquaculture Research.

[6] Evans DH (1980). Kinetic studies of ion transport by fish gill
epithelium. Am J Physiol.

[7] Evans DH, Piermarini PM, Potts WTW (1999). Ionic transport
in the fish gill epithelium. J Exp Zool.

[8] Ayson FG, Kaneko T, Hasegawa S, Hirano T (1994). Development
of mitochondrion-rich cells in the yolk-sac
membrane of embryos and larvae of tilapia (Oreochromis
mossambicus) in fresh water and seawater. J Exp Zool.

[9] Van der Heijden AJ, Van der Meij JC, Flik G, Wendelaar Bonga SE (1999). Ultrastructure and distribution
dynamics of chloride cells in tilapia larvae in fresh
water and sea water. Cell Tissue Res.

[10] Wales W, Tytler P (1996). Changes in chloride cell distribution
during early larval stages of Clupea harengus. J Fish Biol.

[11] Little EE, Fabacher DL (1994). Comparative sensitivity of
rainbow trout and two threatened salmonids, Apache
trout and Lahontan cutthroat trout, to ultraviolet-B radiation. Arch Hydrobiol.

[12] Olson MH, Colip MR, Gerlach JS, Mitchell DL (2006). Quantifying
ultraviolet radiation mortality risk in bluegill larvae:
effects of nest location. Ecol Appl.

[13] Armstrong TN, Reimschuessel R, Bradley BP (2001). DNA
damage, histology changes and DNA repair in larval
Japanese medaka (Oryzias latipes) exposed to
ultraviolet-B radiation. Aquat Toxicol.

[14] Lesser MP, Barry TM (2003). Survivorship, development and
DNA damage in echinoderm embryos and larvae exposed
to ultraviolet radiation (290-400nm). J Exp Marine
Biol Ecol.

[15] Serrano A, Anton M, Cancillo ML, Mateos VL (2006). Daily
and annual variations of erythemal ultraviolet radiation
in Southwestern Spain. Ann Geophys.

[16] Steeger HU, Freitag JF, Michl S, Wiemer M, Paul RJ (2001). Effects of UV-B radiation on embryonic, larval and
juvenile stages of North Sea plaice (Pleuronectes
plates sa) under simulated ozone-hole conditions. Helgol Mar Res.

[17] Applegate LA, Ley RD (1988). Ultraviolet radiation-induced
lethality and repair of pyrimidine dimers in fish embryos. Mutat Res.

[18] Sharma JG, Chakrabarti R (2006). Effects of UV-B radiation
on the gills of Catla catla during early development. Toxicol Environ Chem.

[19] Hakkinen J, Pasanen S, Kukkonen JVK (2000). The effect of
solar UV-B radiation on embryonic mortality and development
in three boreal anurans (Rana temporaria,
Rana arvalis and Bufo bufo). Chemosphere.

[20] Hakkinen J, Oikari A (2004). A field methodology to study
effects of UV radiation on fish larvae. Water Res.

[21] Hakkinen J, Vehniainen E, Ylonen O, Heikila J, Soimasua M, Karuole J (2002). The effects of increasing
UVB radiation on pigmentation, growth and survival
of coregonid embryos and larvae. Environ Biol fishes.

[22] Flamarique IN, Harrower WL (1999). Mortality of sockee
salmon raised under light backgrounds of different
spectral composition. Environ Biol Fishes.

[23] Kaweewat K, Hofer R (1997). Effect of UV-B radiation on
goblet cells in the skin of different fish species. J Photochem
PhotobiolB.

[24] Noceda C, Gonzalez-Sierra S, Martinez JL (1997). Histopathology
of UV-B irradiated brown trout (Salmo trutta)
skin. Dis Aquat Org.

[25] Beland F, Browman HI, Rodriguez CA, Jean-Francois STP (1999). Effect of Solar Ultraviolet radiation (280-
400nm) on the egg and larvae of Atlantic cod (Gadus
morhua). Can J Fish Aquatic Sci.

[26] Ghanizadeh Kazerouni E, Khodabandeh S (2010). Effects of
ultraviolet radiation on skin structure and ultrastructure
in the Caspian Sea Salmon, Salmo trutta caspius,
during alevin stage. Toxicol Environ Chem.

[27] Bullock AM, Roberts RJ (1992). The influence of ultraviolet-B
radiation on the mechanism of wound repair in the
skin of the Atlantic salmon, Salmo salar L. J Fish Dis.

[28] Sharma JG, Masuda R, Tanaka M (2005). Ultrastructural
study of skin and eye of UV-B irradiation ayu, Plecoglossus
altivelius (Pisces, Family: Plecoglossidae). J Fish Biol.

[29] Bullock AM, Coutts R (1985). The impact of solar ultraviolet
radiation upon the skin of rainbow trout, Salmo gairdneri
Richardson, farmed at high altitude in Bolivia. J Fish Dis.

[30] Walter C, Ward B (1998). Is solar radiation responsible for
decline in marine survival rate of anadromous salmonids
that rear in small streams?. Can J Fish Aquat Sci.

[31] Kazanchev EN, Shariati A (1992). Fishes of the Caspian Sea and its
drainage basin.

[32] IUCN (1996). The red list of threatened animals.

[33] Khodabandeh S, Kutnic M, Aujoulat F, Charmatier G, Charmantier-Danures M (2005). Ontogeny of the antennal
gland in the crayfish Astacus leptodactylus
(Crastacean, Decapoda): immunolocalization of
Na^+^/K^+^-ATPase. Cell Tissue Res.

[34]  Khodabandeh S, Charmatier G, Blasco C, Grousset E, Charmantier-Danures M (2005). Ontogeny of the antennal
gland in the crayfish Astacus leptodactylus (Crastacean,
Decapoda): anatomical and cell differentiation. Cell Tissue Res.

[35] Varsamos S, Diaz JP, Charmantier G, Blasco C, Connes R, Flik G (2002). Location and morphology of chloride
cells during the postem bryonic development of
the European sea bass, Dicentrarchus labrax. Anat Embryol.

[36] Hwang PP (1989). Distribution of chloride cells in teleost larvae. J Morphol.

[37] Fischer-Scherl T, Hoffmann RW (1988). Gill morphology of
native brown trout Salmo trutta m. fario experiencing acute and chronic acidification of a brook in Bavaria,
FRG. Dis aquat Org.

[38] Mallat J (1985). Fish gill structural changes induced by toxicants
and other irritants: a statistical review. Can J
Fish Aquat Sci.

[39] Parashar RS, Banerjee TK (2002). Toxic impact of lethal concentration
of lead nitrate on the gills of air-breathing
catfish Heteropneustes fossilis (Bloch). Veterinarski
Arhiv.

